# Conformational Changes of Glutamine 5′-Phosphoribosylpyrophosphate Amidotransferase for Two Substrates Analogue Binding: Insight from Conventional Molecular Dynamics and Accelerated Molecular Dynamics Simulations

**DOI:** 10.3389/fchem.2021.640994

**Published:** 2021-02-26

**Authors:** Congcong Li, Siao Chen, Tianci Huang, Fangning Zhang, Jiawei Yuan, Hao Chang, Wannan Li, Weiwei Han

**Affiliations:** ^1^ Key Laboratory for Molecular Enzymology and Engineering of Ministry of Education, Engineering Laboratory for AIDS Vaccine, School of Life Science, Jilin University, Changchun, China; ^2^ Jilin Province TeyiFood Biotechnology Company Limited, Changchun, China

**Keywords:** 5′-phosphoribosylpyrophosphate amidotransferase, substrates analogue, molecular dynamics simulations, accelerated molecular dynamics simulations, conformational changes

## Abstract

Glutamine 5′-phosphoribosylpyrophosphate amidotransferase (GPATase) catalyzes the synthesis of phosphoribosylamine, pyrophosphate, and glutamate from phosphoribosylpyrophosphate, as well as glutamine at two sites (i.e., glutaminase and phosphoribosylpyrophosphate sites), through a 20 Å NH_3_ channel. In this study, conventional molecular dynamics (cMD) simulations and enhanced sampling accelerated molecular dynamics (aMD) simulations were integrated to characterize the mechanism for coordination catalysis at two separate active sites in the enzyme. Results of cMD simulations illustrated the mechanism by which two substrate analogues, namely, DON and cPRPP, affect the structural stability of GPATase from the perspective of dynamic behavior. aMD simulations obtained several key findings. First, a comparison of protein conformational changes in the complexes of GPATase–DON and GPATase–DON–cPRPP showed that binding cPRPP to the PRTase flexible loop (K326 to L350) substantially effected the formation of the R73-DON salt bridge. Moreover, only the PRTase flexible loop in the GPATase–DON–cPRPP complex could remain closed and had sufficient space for cPRPP binding, indicating that binding of DON to the glutamine loop had an impact on the PRTase flexible loop. Finally, both DON and cPRPP tightly bonded to the two domains, thereby inducing the glutamine loop and the PRTase flexible loop to move close to each other. This movement facilitated the transfer of NH3 via the NH3 channel. These theoretical results are useful to the ongoing research on efficient inhibitors related to GPATase.

## Introduction

**FIGURE 1:**
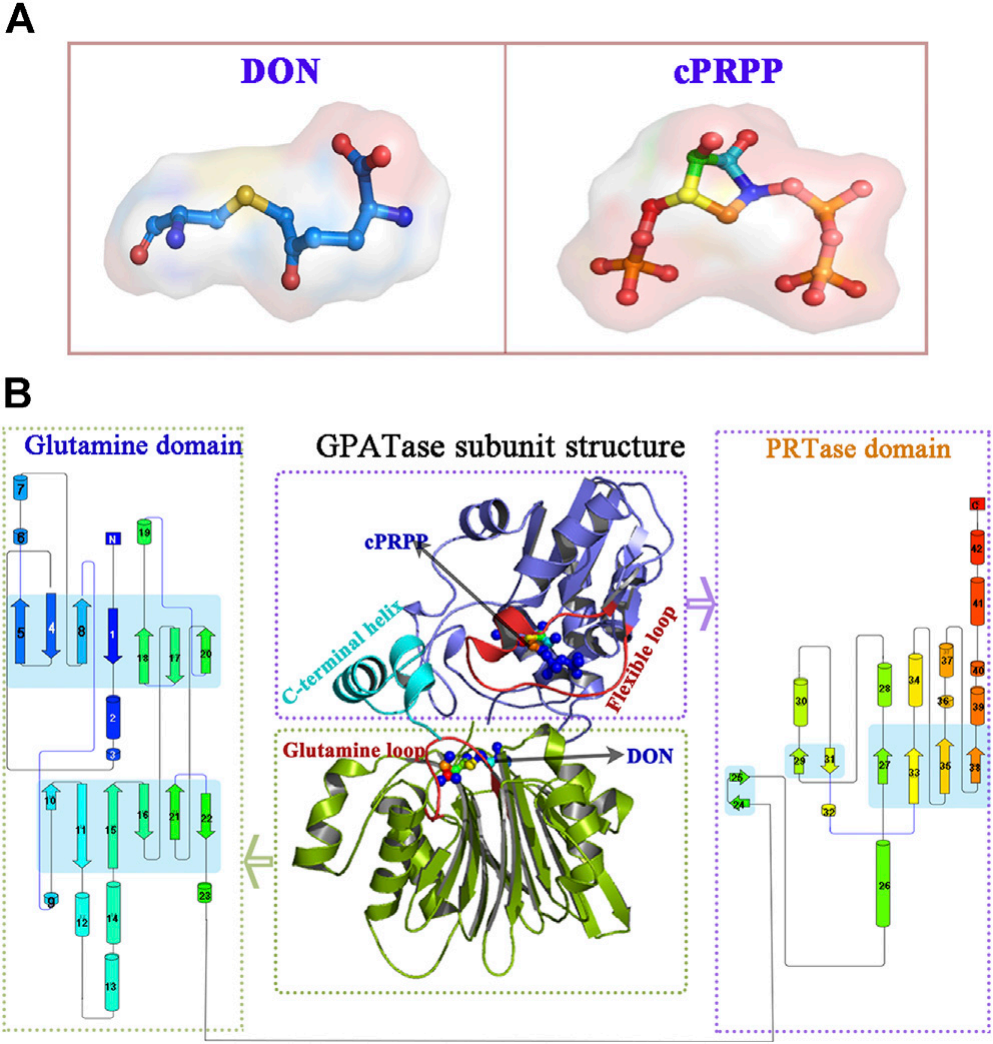
The overview of GPATase structure and the ligands. **(A)** The stereochemical structure of DON and cPRPP; **(B)** GPATase subunit structure and topological structure of the protein in the glutamine domain and PRTase domain made by Pro-origami.

GPATase catalyzes the first step of *de novo* purine nucleotide synthesis and takes the sum of two ‘half reactions’ at different active sites in separate domains (i.e., the Gln and the PRTase domains) (Krahn et al., 1997; Wolan et al., 2004; Wang et al., 2009; Fan et al., 2019; Cao et al., 2020). It is a key enzyme in the total synthetic pathway, and it transfers nitrogen from glutamine to phosphoribosylpyrophosphate (PRPP) (Chittur et al., 2001; Zhu et al., 2017; Semmelmann et al., 2019). This enzyme consists of both a glutaminase domain and a synthase domain. The glutamine domain in its N-terminal is an Ntn hydrolase in conserved enzyme groups (Brannigan et al., 1995; Teplyakov et al., 1999). Its C-terminal domain is a phosphoribosyltransferase (PRTase) (Musick and Nyhan, 1981) (Figure 1).

GPATase is a type I PRTase (Grubmeyer et al., 2012). Several crystal structures of type I PRTases (Krahn et al., 1997; Singh and Christendat, 2006; González-Segura et al., 2007; Fan et al., 2009; Xiang et al., 2009; Lund et al., 2010; Kang et al., 2012) have been determined. Ammonia is transferred by the ammonia channel, and this process depends on the active sites of the two domains. However, two questions must be answered. How do the two domains mutually effect each other? What causes conformational changes in GPATase? Recently, enhanced sampling molecular simulations have been used to successfully probe the effect of multisite PTMs on the mobility of several regions for HMGB1-PtDNA complexes (Lyu et al., 2018). Consequently, cMD simulations and aMD simulations were performed to elucidate the changes in protein structures that occur when substrate analogues bind to GPATase. The systems and all molecular dynamic simulations performed in this work are summarized in Table 1.

**TABLE 1:** Summary of molecular dynamics simulations performed in this work.

	System	cMD (ns)	aMD (ns)
1	GPATase	200	400
2	GPATase–DON	200	400
3	GPATase–cPRPP	200	400
4	GPATase–DON-cPRPP	200	400

cMD simulations as an efficient tool are increasingly used to probe the structural biology of biomolecules (Wang et al., 2018; Zhu et al., 2020). However, cMD simulations have difficulty capturing numerous conformational changes because of several limitations. Accelerated molecular dynamics (aMD) (Hamelberg et al., 2007; Kappel et al., 2015) applies a bias potential so that researchers can not only monitor conformational changes more efficiently than cMD but also use fewer computing resources (Tsai et al., 1999; Gasper et al., 2012; Spindel, 2012). More importantly, this technology has achieved success in probing proteins conformational changes induced by ligand bindings (Wang et al., 2016; Kokh and Amaral, 2018; Peng et al., 2018; Wang and Miao, 2019). For example, Grant *et al.* employed aMD simulations to probe the GTP and GDP modified structure changes of Ras proteins, their findings suggest that aMD can capture nucleotide dependent conformational switching but cMD simulations cannot, showing the advantages of aMD simulations. In this study, we first performed cMD to observe the effect of two substrate analogues (i.e., DON and cPRPP) on the structural stability of GPATase. Despite the impact of different ligand binding modes on the conformational change of two active sites being studied by cMD simulations, it is hard to capture how the conformational transition process induced by two domains mutually effect each other. Therefore, to realize our aims, performing aMD simulations could provide detailed pictures of the conformational changes in the Glutamine loop and PRTase flexible loop of the enzymes. Moreover, it can describe the mechanism of the synergistic actions of these two domains.

## Materials and Methods

### System Setup

The coordinates of *E. coli* GPATase (PDB code 1ECC) (Krahn et al., 1997) complexed with cPRPP and DON were downloaded from the Protein Data Bank (Figure 1). As the initiating structure, cPRPP was used as a nonhydrolyzable analogue of PRPP substrate. In this crystalline structure, the side chain of Cys1 is covalently modified by DON, which is a glutamine affinity analog (Krahn et al., 1997; Parry et al., 1997). Four models were constructed: 1) GPATase without ligands, 2) only DON remained in the GPATase (Mn^2+^ was coordinated by O@water in Model 1 and Model 2), 3) only cPRPP remained in the GPATase (Mn^2+^ was ligated by O1, O2, O3, O1β@cPRPP, and O@water), and 4) GPATase with two ligands (this model is a combination of Model 2 and Model 3). MCPB. py (Li and Merz, 2016) (version 3.0, released in AmberTools 17) was utilized to build force fields based on quantum calculations and simulate metal ions containing coordination compounds via the bonded model approach (Cornell et al., 1995). The Mn^2+^ parameters used in this program were from Li et al. (Li et al., 2013). All models were prepared using the Leap module from the AMBER16 suite and were characterized by employing the ff14SB force field (Maier et al., 2015). All histidine residues were protonated at the ε position. All missing hydrogen atoms were added to cPRPP using the Leap program from the AMBER16 package (Götz et al., 2012). The geometries of cPRPP were refined with Gaussian16 at the B3LYP/6-31G* level (Slanina et al., 2004). The optimized geometries were used to calculate charges derived from electrostatic potential derived (ESP) following the RESP methodology (Cornell et al., 1993) implemented in the antechamber module in Amber16. The neutral charge of the systems was maintained by adding 9, 11, 12, and 15 Na^+^ ions respectively to Model 1, Model 2, Model 3, and Model 4. Each model was solvated in a cubic box with a buffer of no less than 12 Å and used TIP3P water molecules to generate water model parameters (Paschek et al., 2011).

### Molecular Dynamics Simulations

Four cMD simulations were performed using the pmemd. cuda module of AMBER16 (Götz et al., 2012). Electrostatic interactions were calculated via the smooth particle mesh Ewald method, and the cut-off distance of non-bonded real space interactions was 10 Å (Darden et al., 1993). The bonds containing hydrogen atoms were restrained, using the SHAKE algorithm, and the integration time step for four simulations was 2 ps (Ryckaert et al., 1977). Inappropriate geometry and spatial conflicts were avoided by minimizing the energy before the cMD simulations via the steepest descent algorithm and conjugate gradient minimizer method (Jie and Liu, 2003). The cMD production for all four systems were ran under constant temperature (310 K) and 1 atm constant pressure conditions (Berendsen et al., 1998). Subsequently, 200 ns cMD simulations were performed for each system with periodic boundary conditions. Saving atomic coordinates every 100 ps for further analysis.

### Accelerated Molecular Dynamics Simulations

aMD is a means of enhancing sampling technology by bridging the potential energy surface. It can overcome the drawbacks of cMD, which has difficulty monitoring domain structure variation and long-distance correlated motions that occur over very long timescales (Hamelberg et al., 2004; Hamelberg et al., 2007; Markwick and Mccammon, 2011). In this study, four dual-boost aMD simulations of GPATase were initiated from the final structure of the corresponding cMD simulations. Before running dual-boost aMD, the following aMD parameters were calculated: 1) EthreshP ($E_{tot}$), which denotes the average total potential energy threshold; 2) alphaP ($Alpha_{tot}$), which is the inverse strength boost factor for the total potential energy; 3) EthreshD ($E_{dih}$), which represents the average dihedral energy threshold; and 4) alphaD ($Alpha_{dih}$), which is the inverse strength boost factor for the dihedral energy. These parameters were calculated using the following formula (Pierce et al., 2012):

$$E_{tot}=E_{tot\;avg}+N_{atoms}\times 0.16$$ (1)

$$Alpha_{tot}=N_{atoms}\times 0.16$$ (2)

$$E_{dih}=E_{dih\;avg}+N_{resid}\times 4$$ (3)

$$Alpha_{dih}=\frac{1}{5}\times \left(4\times N_{resid}\right)$$ (4)

where $E_{tot\;avg}$ and $E_{dih\;avg}$ are obtained from cMD simulations. $N_{atoms}$ is the number of the whole system atoms, and $N_{resid}$ is the total number of residues. For each system, 400 ns aMD simulations were performed and aMD parameters settings are based on cMD simulation results.

### Subnetwork Analysis of Protein-Ligand Complexes

Protein structure networks offer invaluable insights into the structural properties, functions, and stability of proteins (Atilgan et al., 2004; Brinda and Vishveshwara, 2005). In this study, RINerator software was utilized to obtain the RINs of GPATase, GPATase–DON, GPATase–cPRPP, and GPATase–DON–cPRPP. RINalyzer was used to perform the RIN analysis (Doncheva et al., 2011), The results were then visualized using Cytoscape (Shannon et al., 2003). Three-dimensional structures were visualized using the PyMOL software (Delano, 2002) and VMD software (Humphrey et al., 1996). Cluster analysis was performed using the K-means clustering algorithm available in CPPTRAJ (Shao et al., 2007). Detailed clustering results for the top six clusters were summarized in Supplementary Tables S1–S4. The centroid structure of the most populated cluster that had the lowest cumulative distance to every other point was selected as the representative structure. The representative structures obtained from cluster analysis of the four complexes were applied to conduct protein-ligand network analysis.

### Cross-Correlation Analysis and Principal Component Analysis

Dynamic cross correlation analysis (DCCA) was conducted using the Bio3D R package version 2.3.0 (Hünenberger et al., 1995; Grant et al., 2006; Skjærven et al., 2014). The scales of the coordinate axis represent the atomic number in the covariance matrix map, whereas the gradation of color displays the extent of atomic motion correlation. The red regions denote the positive correlation between atoms reflecting motion along the same direction, whereas the blue regions show that the corresponding two atomic motion modes are negatively correlated. PCA was performed using Bio3D (version 2.3.0) and visualized by VMD (version 1.9.3) (Grant et al., 2006; Skjærven et al., 2014). The energy of protein conformations for the first two principal components (PC1 and PC2) were characterized in Free Energy Landscape (Hünenberger et al., 1995). The trajectories to PC1 and PC2 of motion were projected by employing the program converting dot distribution to probability distribution (ddtpd) (Nicolaï et al., 2013; Iida et al., 2016).

## Results and Discussion

### Structural Stability Analysis during cMD Simulations

**FIGURE 2:**
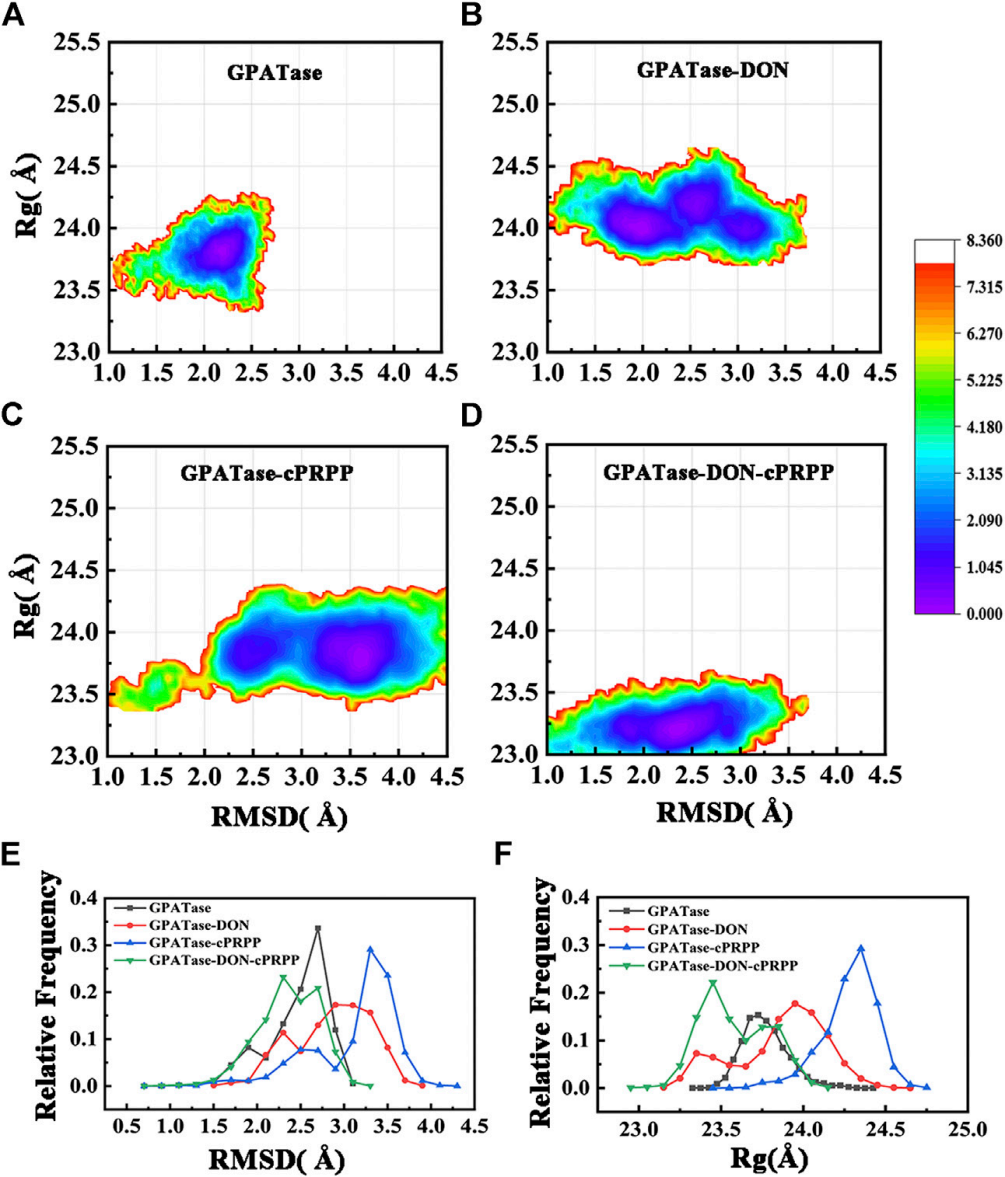
The free energy landscape for *Rg* and RMSD of **(A)** GPATase, **(B)** GPATase–DON, **(C)** GPATase–cPRPP, and **(D)** GPATase–DON–cPRPP. **(E)** Relative frequency of RMSD of the four systems, **(F)** relative frequency of *Rg* of the four systems.

The structural stability of the four complexes was analyzed. The root-mean-square deviation (RMSD) of the protein Cα atom backbone was calculated to illustrate conformational changes. The radius of gyration (Rg) can reflect the plasticity potential of protein structures. The free energy landscapes for both the Rg and RMSD of the four systems are shown in Figures 2A–D. The DON–cPRPP–GPATase system exhibited the main energy basin located at the RMSD value of ∼2.2 Å and the *R*_*g*_ value of ∼23.4 Å, both of which were smaller than those of the three other systems. The corresponding probability of RMSD and *R*_*g*_ of the four systems is displayed in Figures 2E,F. The four systems achieved convergence during the cMD simulations (Supplementary Figure S1A). The RMSDs of the GPATase without ligand and the GPATase with two substrate analogue bonds reached equilibrium at 2.6 Å after 130 ns. By comparison, the RMSDs of the GPATase with DON reached equilibrium at 3.2 Å after 175 ns. The Rg value of the GPATase-DON-cPRPP system stabilized at about 23.8 Å, whereas the *R*_*g*_ value of the enzymes with DON or cPRPP was larger, indicating that the GPATase–DON–cPRPP structure was more compact than others (Supplementary Figure S1B). These results demonstrated that the GPATase with two substrate analogues was more stable than those with DON or cPRPP only.

**FIGURE 3:**
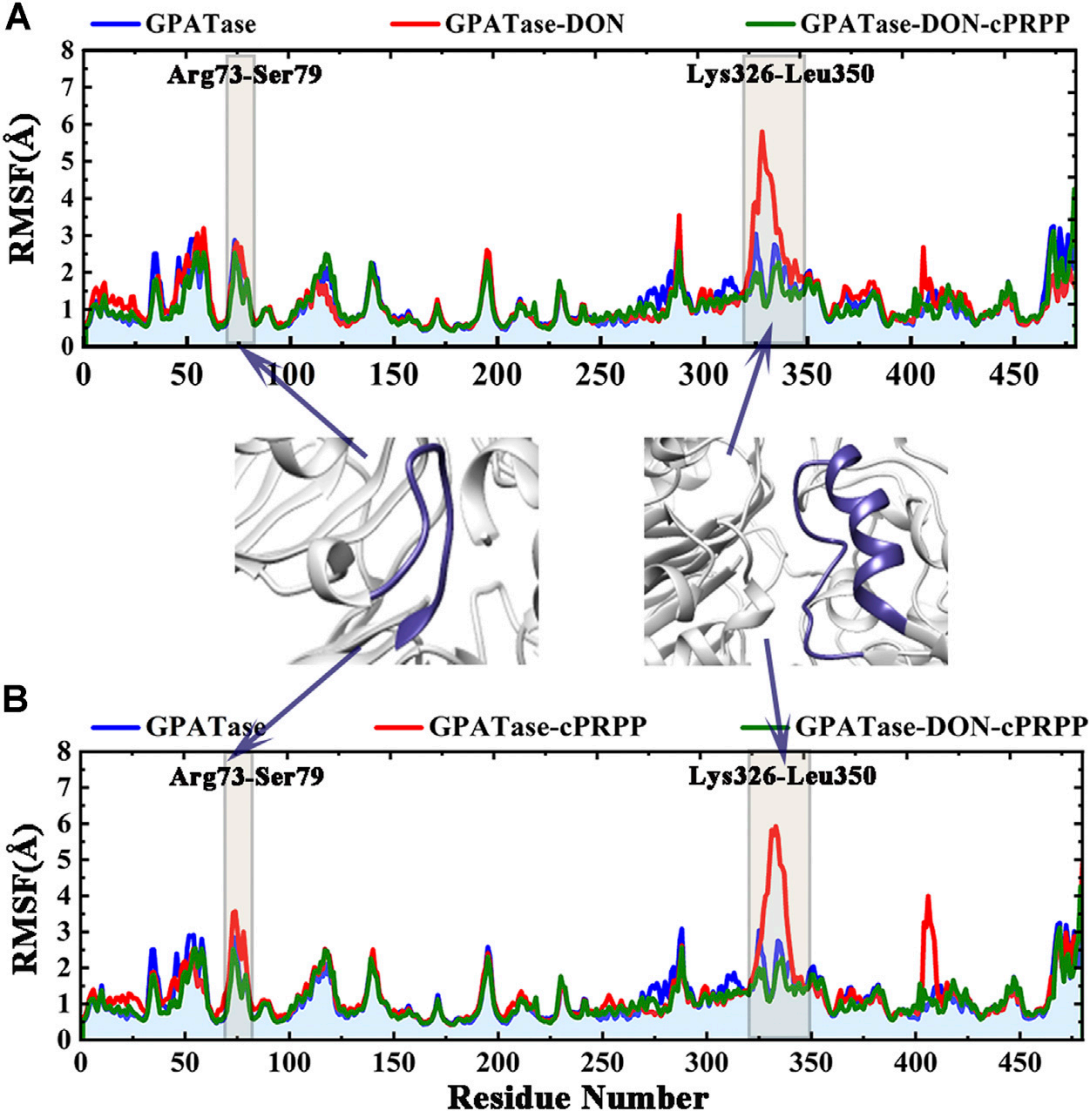
RMSF values of the backbone atoms of the four systems over time during the simulations. Comparison of the RMSF plots of proteins in **(A)** GPATase, GPATase–DON, GPATase–DON- cPRPP complexes; **(B)** GPATase, GPATase–cPRPP, GPATase–DON- cPRPP complexes.

**FIGURE 4:**
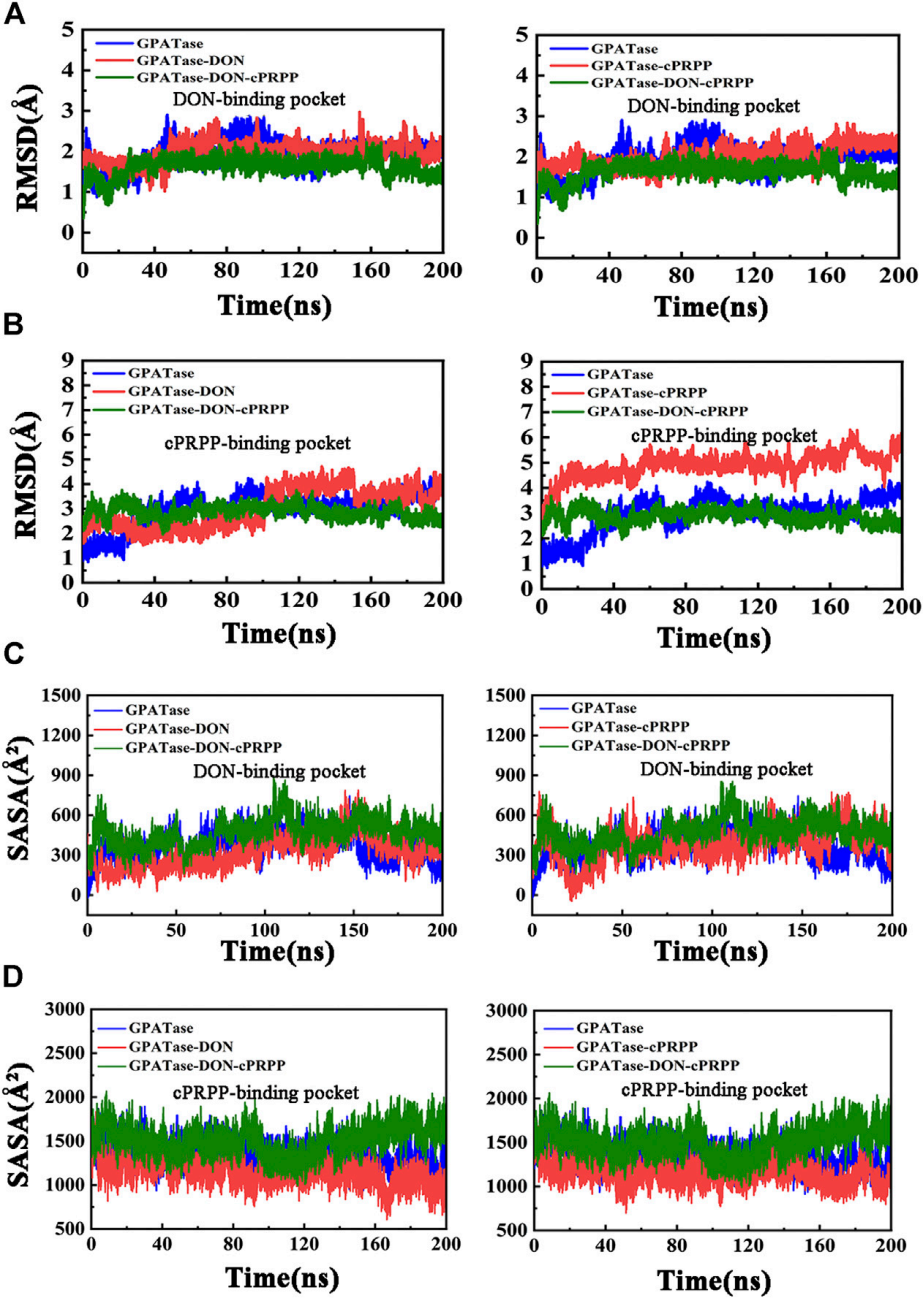
Stability analysis of DON binding site and cPRPP binding site for systems during the 200 ns simulations. **(A)** The RMSD values of DON binding site, **(B)** RMSD plot of cPRPP binding site of the four systems during 200 cMD simulations. **(C)** SASA plot for DON binding pocket. **(D)** SASA plot for cPRPP binding pocket.

**FIGURE 5:**
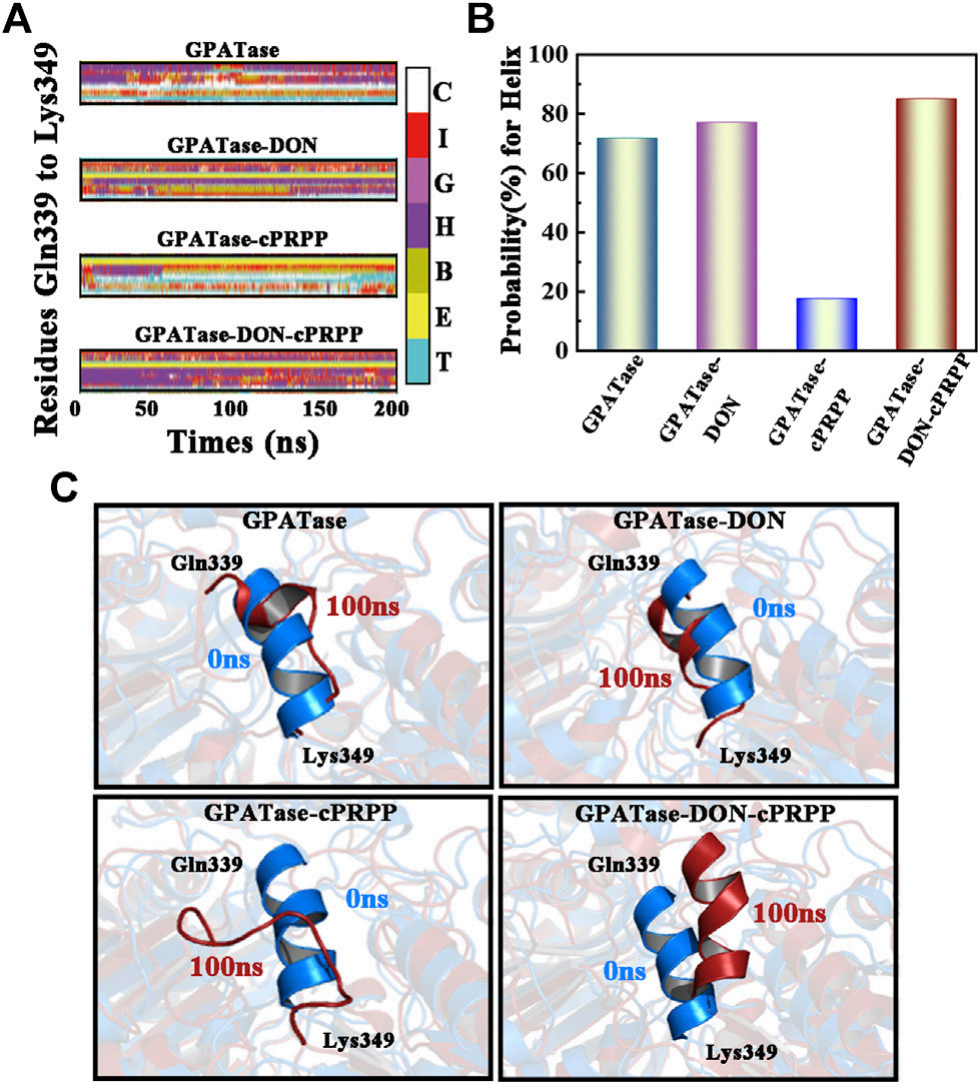
Analysis of changes in secondary structures. **(A)** DSSP results of the four systems, the color bar represented different secondary structures. **(B)** The probability of α-helix (Gln339-Lys349) during 200 cMD simulations. **(C)** The corresponding cartoon structure of the 0 and 100 ns cMD simulations for the four systems.

Root-mean-square fluctuation (RMSF) was used to compute the fluctuations of a single residue. RMSF values can display the extent of freedom for atomic motion, indicating the flexibility of a protein region. RMSF values were calculated at equilibration state. The glutamine active site domain (R73-S79) and the PRTase active site domain (K326-L350) are highlighted in Figures 3A,B. The RMSF values fluctuated, and typical conformations are also displayed in the cartoon. The corresponding RMSD values during the 200 ns cMD simulation of R73-S79 and K326-L350 are shown in Figures 4A,B. The solvent accessible surface area (SASA) contribution of the active binding pocket (i.e., the glutamine and the PRTase active sites) of the GPATase–DON–cPRPP complex was the greatest among the four systems, indicating that this complex could provide a decent hydrophilic environment for ligand binding (Figures 4C,D). Changes in the secondary structures of the complexes, primarily those in Q339 to K349 (which are a part of the PRTase active site domain), are plotted in Figure 5A. The α-helix (Q339 to K349) of the GPATase bound to DON and cPRPP was almost contained, whereas that of the GPATase with cPRPP completely disappeared (Figure 5B). In the two other systems, the α-helix partly disappeared. The corresponding cartoon structures during the 0 and 100 ns cMD simulations of the four systems are exhibited in Figure 5C. Results of cMD analysis revealed mutual stabilization between DON and cPRPP, and they exerted a synergistic effect on the stability of the enzymes.

### Cross Correlations and PCA Calculations

**FIGURE 6:**
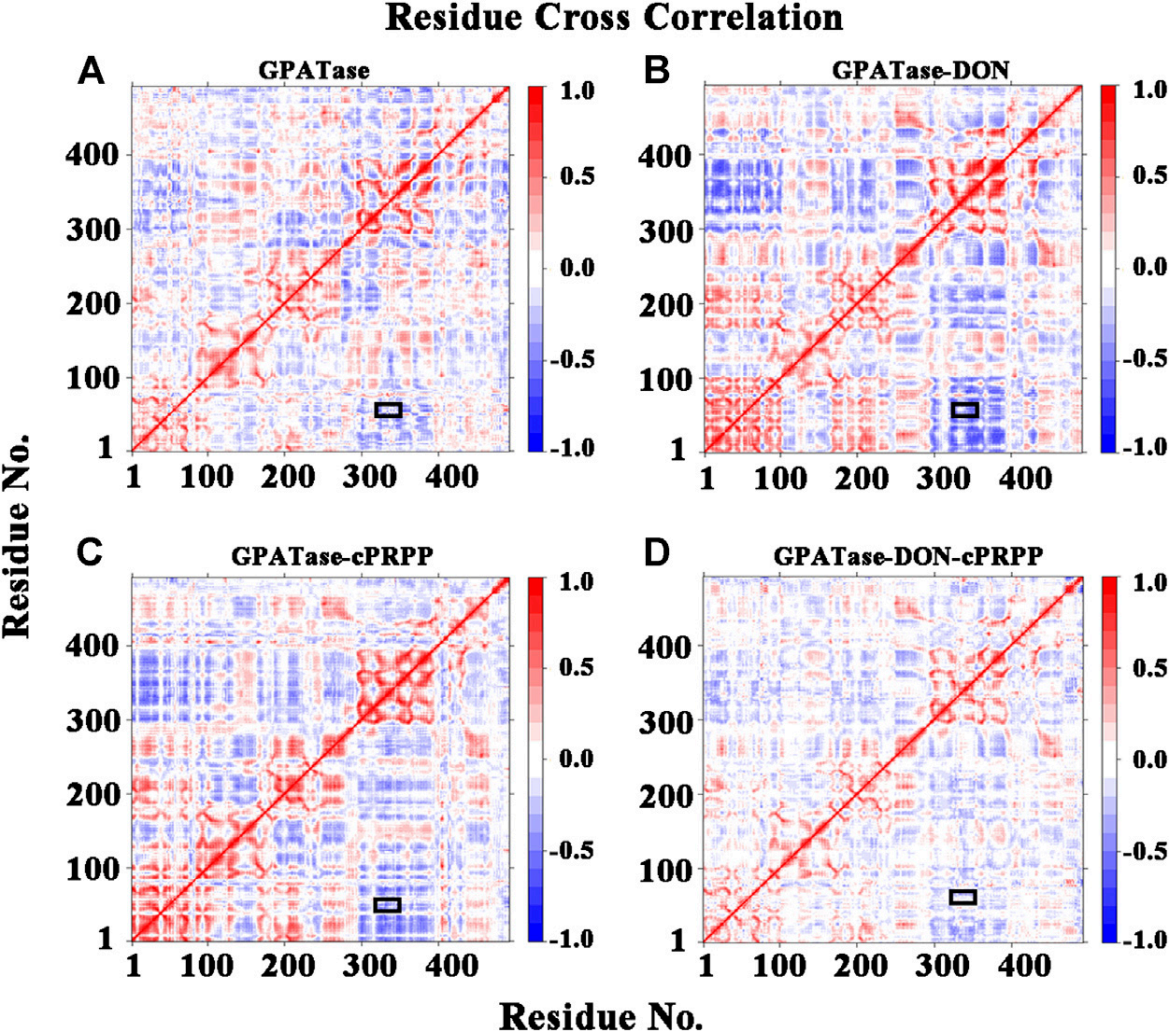
The residues cross-correlation maps for four systems **(A)** GPATase, **(B)** GPATase–DON, **(C)** GPATase–cPRPP, and **(D)** GPATase–DON- cPRPP. The positions of glutamine loop and flexible loop were labeled by black boxes.

DCCA values were computed using cMD trajectories at the equilibrium state to explore the internal dynamics of GPATase. Cross-correlation maps of correlated intermolecular motions between the remote regions of the proteins in different complexes are presented in Figures 6A–D. GPATase with DON or cPRPP displayed larger fluctuations than the two other systems. The GPATase–DON–cPRPP complex manifested the lightest color in the cross-correlation matrix maps, indicating that it experienced the weakest flexibility and it was the most stable during the simulations. Furthermore, the correlation analysis revealed negative correlation between the RTase flexible loop (K326 to L350) and the glutamine loop (R73 to E84) (the relevant regions are highlighted by black boxes). The binding of DON and cPRPP to the GPATase–DON–cPRPP complex weakened the negative correlation between the two loop domains.

**FIGURE 7:**
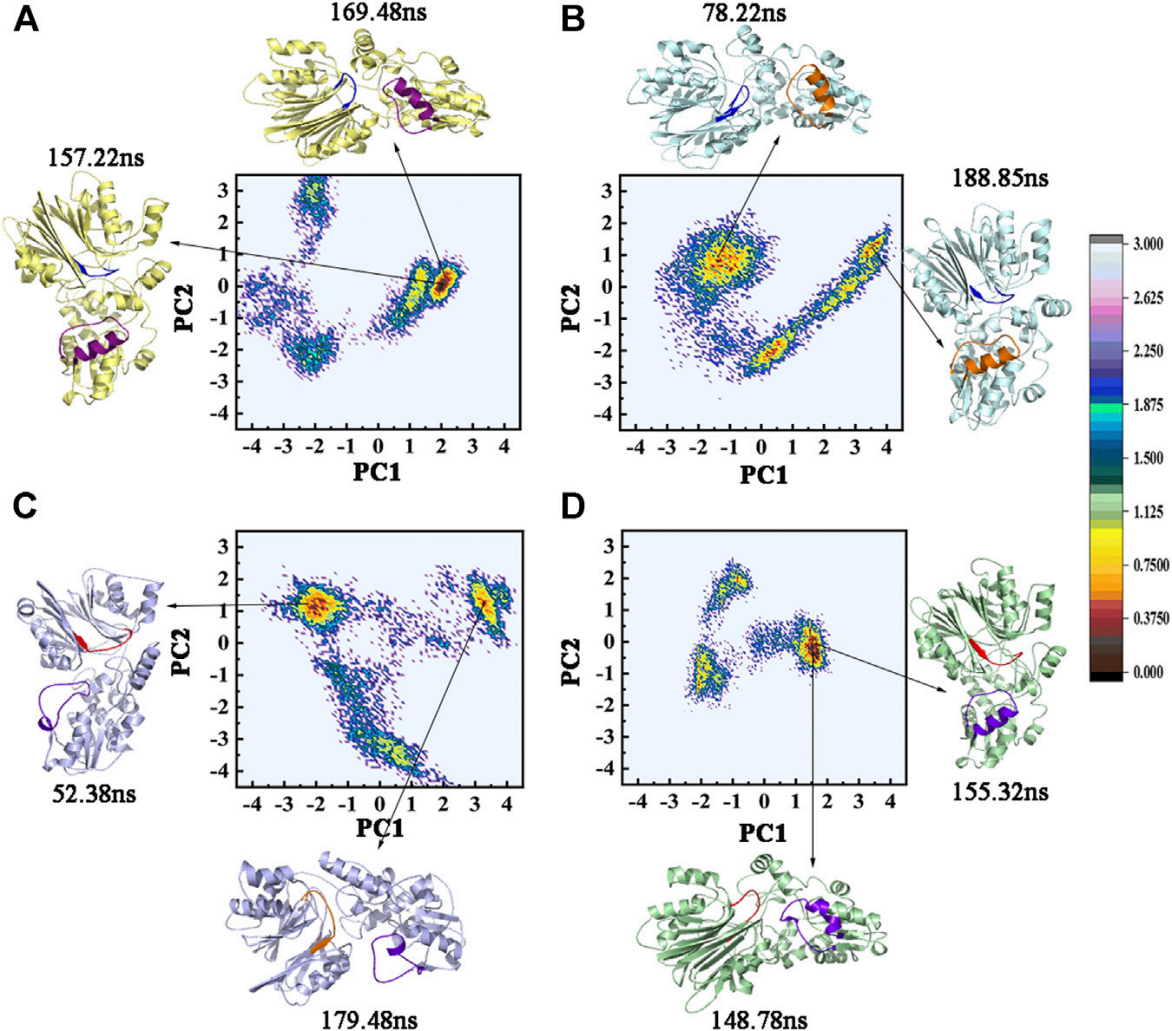
The two-dimensional projection of total cMD conformational space on the first two principal components (PC1 and PC2). FEL maps and PC1 and PC2 structures for **(A)** GPATase, **(B)** GPATase–DON, **(C)** GPATase–cPRPP, and **(D)** GPATase–DON–cPRPP systems. The conformation of GPATase proteins with one global minimum is marked in red. The depth of the energy landscape indicates the value of the minimum free energy.

The dominant conformational changes were identified via PCA analysis of the Ca atoms in the cMD trajectories. The overall motion was determined via PCA through the eigenvectors of the covariance matrix. The two-dimensional projections of the total cMD conformational space on the first two principal components, namely, PC1 and PC2, are given in Figures 7A–D. The sum percentage of the first two principal components (PC1 and PC2) in the four systems accounted for about 50%, which ensured the dependability of the observed motions (Table 2). The conformations of the lowest and the sub-lowest energies shown in Figure 7 indicated that the two active sites had different movement trends. The low-energy regions of the GPATase–DON–cPRPP complex were larger and more centralized than those of the GPATase complexes with only DON or cPRPP. This phenomenon also explained why the latter reached equilibrium after a long time during the MD dynamic simulations. This result was consistent with that of RMSD analysis. These observations implied that the presence of different ligands produced varying effects on the dynamic behavior of GPATase.

**TABLE 2:** Principle component probability during cMD simulations.

Protein	Principle component (PC)	Probability (%)
GPATase	PC1	35.85
	PC2	16.04
GPATase–DON	PC1	34.85
	PC2	14.24
GPATase–cPRPP	PC1	30.57
	PC2	22.30
GPATase–DON–cPRPP	PC1	38.07
	PC2	12.71

### Subnetwork Analysis of Protein-Ligand Interaction

**FIGURE 8:**
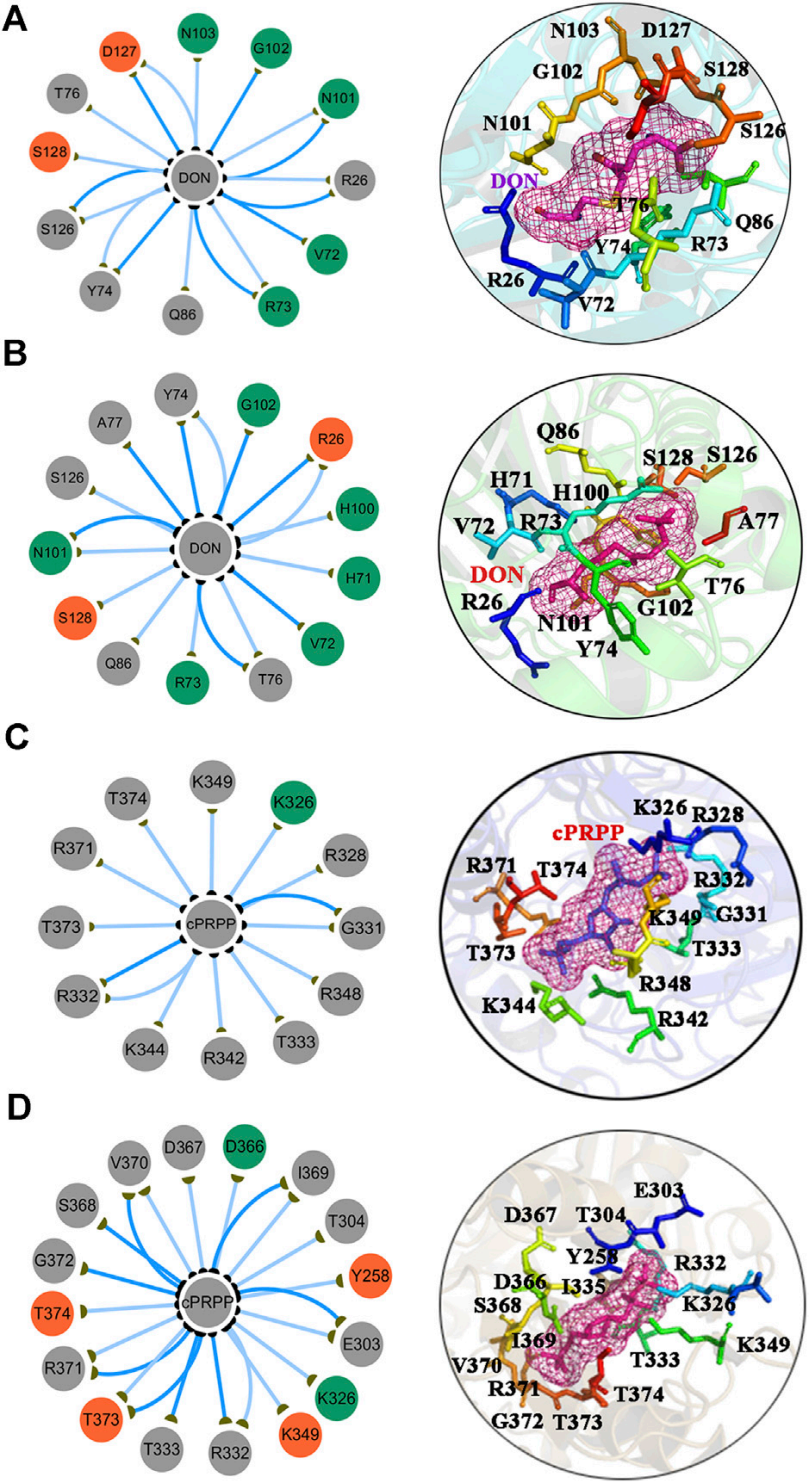
The subnetwork interaction analysis between ligands and protein. The subnetwork between GPATase and DON in the **(A)** GPATase–DON complex and **(B)** GPATase–DON- cPRPP complex. The subnetwork between GPATase and cPRPP in the **(C)** GPATase–cPRPP complex and **(D)** GPATase–DON- cPRPP complexes.

The subnetworks of the interfaces between the ligands and the proteins were extracted to understand differences in the binding affinity of DON and cPRPP to the complexes (Figures 8A–D). The interfaces suggested that the synergism of DON and cPRPP may strengthen the interactions between the ligands and the protein. Thus, the number of interactions in the GPATase–DON–cPRPP complex was greater than that in the DON or cPRPP binding complexes. This result was further verified by analyzing the hydrogen bonds between the ligands (DON or cPRPP) and GPATase in the three complexes during the cMD simulations. The probabilities of hydrogen bond formation are listed in Table 3. The probability of hydrogen bond formation was higher between the DON/cPRPP and GPATase in the DON–cPRPP–GPATase complex than that in the DON- or cPRPP-bound complexes. These results indicated that the combination of cPRPP and DON may induce more stable interaction between the ligands and GPATase, as well as an inseparable communication between the nodes in the glutamine and the PRTase active sites.

**TABLE 3:** Probability of hydrogen bond formation between protein (GPATase) and ligands (DON and cPRPP) for the GPATase–DON, GPATase–cPRPP, and GPATase–DON-cPRPP structures during the 200 ns cMD simulations.

System	Donor	Receptor	Occupancy (%)
GPATase–DON	Arg73:NE	DON:OXT	20.82
	Arg73:NH2	DON:O1	21.42
	Arg73:NH2	DON:OXT	43.35
	Ala77:N	DON:O1	15.51
	Thr76:N	DON:O1	13.91
GPATase–DON-cPRPP	Arg73:NE	DON:OXT	51.68
	GLY102:N	DON:OD	17.84
	Arg73:NH2	DON:OXT	71.74
	Arg73:NH	DON:O1	83.23
	THR76:N	DON:OXT	60.61
	THR76:N	DON:O1	62.02
GPATase–cPRPP	Arg342:NH1	cPRPP:O3	65.20
	Lys349:NZ	cPRPP:O8	62.55
	Lys344:NZ	cPRPP:O3	66.23
	Arg342:NH2	cPRPP:O3	67.27
	Lys349:NZ	cPRPP:O2	73.52
GPATase–DON-cPRPP	Arg371:NH1	cPRPP:O6	67.50
	Thr333:N	cPRPP:O9	83.03
	Thr333:N	cPRPP:O8	64.01
	Phe334:N	cPRPP:O9	62.62
	Thr258:OH	cPRPP:O9	98.61
	Thr333:OG1	cPRPP:O8	98.93
	Lys349:NZ	cPRPP:O1	84.73
	Thr374:OG1	cPRPP:O3	94.58
	Arg343:NH1	cPRPP:O5	70.75

The previous studies indicated that NH_3_ can transfer between two active sites without external potential in GPATase by performing LES/PMF simulations (Xiang et al., 2009), so two domains mutually effecting each other induced by substrate binding was likely to be the main factor to facilitate ammonia travel. We performed the above cMD simulations to further decode atomic level mechanism relating to structure changes of GPATase. The cMD analysis results provide a basis for further performing aMD simulations.

### The Stability Analysis of R73-DON Salt Bridge during aMD Simulations

**FIGURE 9:**
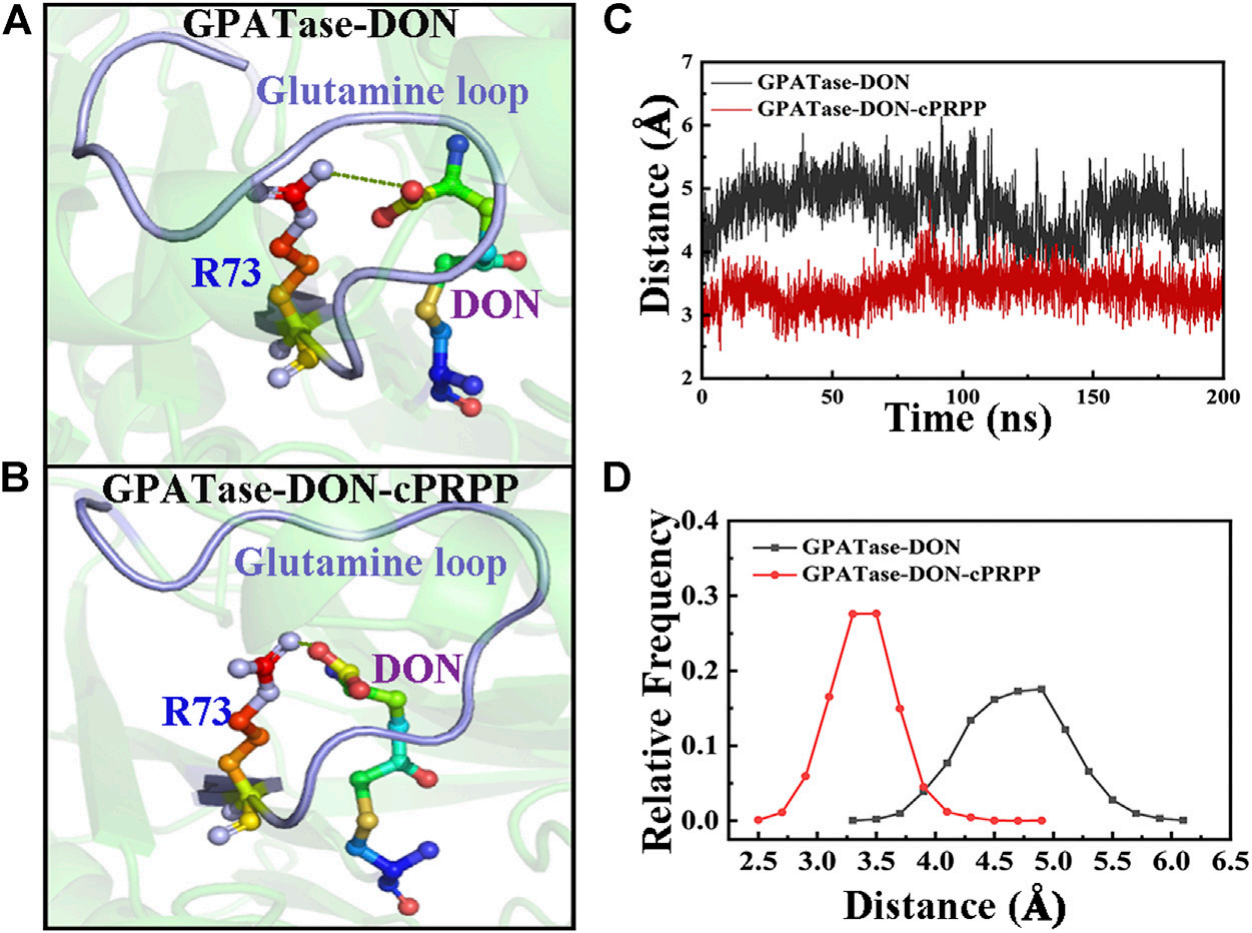
Comparison of R73–DON salt bridges between the GPATase–DON and GPATase–DON–cPRPP complexes during aMD simulations. The salt bridges are shown as green dashed lines. The glutamine loop is marked in light blue. R73 and DON are presented as sticks. Representative structures of the **(A)** GPATase–DON and **(B)** GPATase–DON–cPRPP complexes. **(C)** Variations in the distance between R73 and DON of the two systems. **(D)** Relative frequency of distance of the two systems.

The conformational states and structural dynamics of GPATase in each system were evaluated via 400 ns aMD simulations. The RMSD values were clearly convergent for the aMD simulations (Supplementary Figure S2). R73 was optimally positioned for binding with glutamine, and the PRTase sites were connected by a 20 Å NH3 channel. R73 formed a salt bridge with the carboxyl group of glutamine analogues, which was improperly positioned for this interaction (Bera et al., 1999). A salt bridge formed between the R73 and DON occurred only in the active conformer of the enzyme. The representative conformations of the GPATase–DON and GPATase–DON–cPRPP are shown in Figures 9A,B, respectively. The ability of cPRPP to bind or not to bind to GPATase and its influence on the formation of salt bridge were explored. The distance between R73 and DON during the 400 ns aMD simulations is given in Figure 9C. A distance of less than 4 Å denotes the formation of a salt bridge (Kumar and Nussinov, 2002). The distance had larger fluctuations for GPATase bound to DON only than the GPATase–DON–cPRPP complex (Figure 9C). Moreover, the peak value for the GPATase–DON complex was 3.2 Å, whereas that for the GPATase–DON–cPRPP complex was 4.8 Å (Figure 9D). According to the results of the subnetwork analysis summarized in Figure 8 and Table 3, the probability of hydrogen bond formation between R73 and DON was higher for the GPATase–DON–cPRPP complex than for the complex without cPRPP. Hence, the hydrogen bond interactions between them moved the side chain of R73 closer to the DON carboxylate group. This movement may be useful to the formation of a salt bridge. Furthermore, salt bridges between R73 and DON carboxylate formed only in the active form of the enzyme, indicating that binding cPRPP to the PRTase flexible loop was important for reconstructing the glutamine loop and inducing GPATase in its closed form (i.e., active state).

### Conformational Free Energy Landscape of PRTase Active Site Domain

In each system, 400 ns aMD simulations were carried out to examine the conformational changes and atomic-level molecular mechanism of GPATase. Free energy profile maps of GPATase were explored according to the extension and changes in the shape of the PRTase flexible loop, both of which were important for GPATase activation. The free energy distribution map of each system was projected along with two collective variables (abscissa and ordinate). CV1 reflected the deviation in the PRTase flexible loop from the crystallographic structure (PDB code 1ECC) (Krahn et al., 1997), which was able to describe the opening-closing state of the PRTase flexible loop. The residues moving outward formed an open conformation of the PRTase flexible loop, which was in its inactive state because the binding cavity in the shape was not complementary. The negative value represents the closed conformation status of the flexible loop, whereas the positive value indicates the open status of the loop. CV2 was the distance between I335 and R342, which were the residues in the PRTase flexible loop and had interactions with cPRPP. The values of CV2 characterized the shape of the PRTase active site domain, in others words, the values helped determine whether there was sufficient space for cPRPP to bind.

**FIGURE 10:**
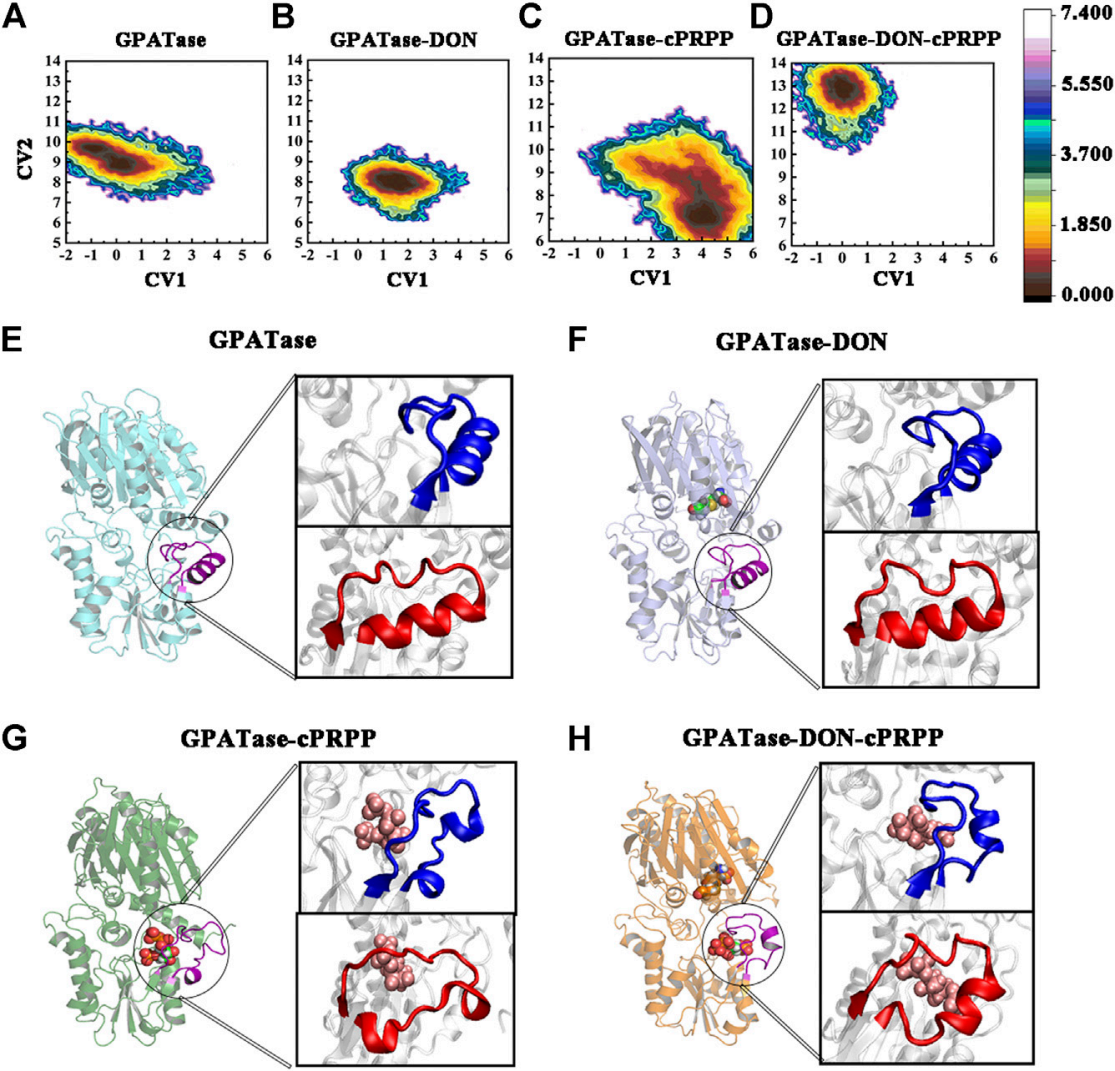
The conformation change of PRTase flexible loops during 400 ns aMD simulations. Free energy profile maps of flexible loops for the **(A)** GPATase; **(B)** GPATase–DON; **(C)** GPATase–cPRPP; and **(D)** GPATase–DON–cPRPP complexes as a function of CV1 and CV2 in Å; representative structures of flexible loops for the **(E)** GPATase; **(F)** GPATase–DON; **(G)** GPATase–cPRPP; and **(H)** GPATase–DON-cPRPP complexes. The PRTase domain active site is shown in cartoon. Cartoons marked in blue and red represent different perspectives.

The free energy profile maps showed remarkable differences in conformational changes in the PRTase flexible loops of each system (Figures 10A–D). To prove the number of aMD samples was sufficient, cMD free energy landscape maps were drawn as a comparison (Supplementary Figure S3), it is obvious that aMD can sample more phase spaces than cMD simulations. As the flexible loop for GPATase with cPRPP binding deviated, the CV1 value sampled in this system mainly distributed between 3 and 5 Å (Figure 10C). The outward deviation suggested that the flexible loop was fully extended and in an open state. A representative conformation is presented in Figure 10G (blue). By contrast, the three other systems were in a closed state (Figures 10E,F,H) (also marked in blue). CV2 fluctuated between 12.5 and 13.5 Å for the GPATase–DON–cPRPP complex. Moreover, the CV2 value of this complex was considerably higher than that of the three other systems, indicating that it had sufficient space to bind cPRPP and it contributed to the enzyme catalytic reaction. The corresponding representative conformations are marked in red in Figures 10E–H. Notably, although the loop status of GPATase bound to DON only was closed, the shape of the active site domain obstructed cPRPP binding because of the relatively small amount of space. By contrast, the PRTase domain of GPATase with DON and cPRPP provided sufficient space for cPRPP binding.

The crystal structures determined by Krahn et al. revealed that the most dramatic structural differences between the inactive and active form of GPATase was its open/close state (Krahn et al., 1997). But dynamics information related to opening-closing conformational transition for the flexible loop remained vague. The above analysis suggested that although the ordering of the PRTase flexible loop was induced by binding cPRPP, the restructuring of the glutamine loop also had a direct steric effect on the PRTase flexible loop that kept it closed and had enough space to assist cPRPP binding to GPATase in the DON–cPRPP–GPATase complex.

### The Relative Position Variation Analysis between I335 and Y74

**FIGURE 11:**
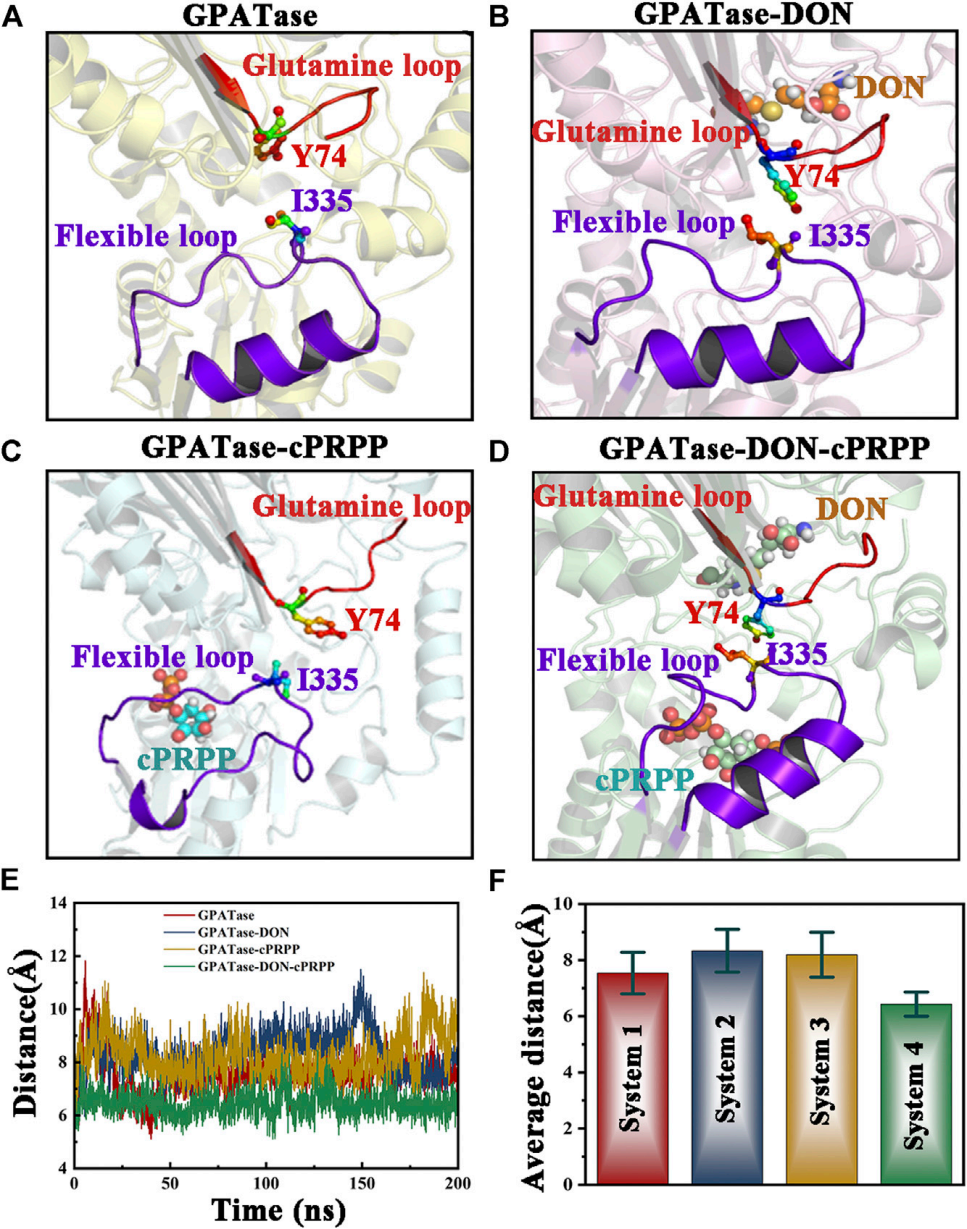
Comparison of the relative position between the glutamine loop and flexible loop of the four systems. Both loops are displayed as cartoons. DON and cPRPP are shown as spheres. Y74 and I335 are depicted as sticks. Representative conformation of the **(A)** GPATase, **(B)** GPATase–DON, **(C)** GPATase–cPRPP, and **(D)** GPATase–DON–cPRPP complexes during aMD simulations. **(E)** Distance between Y74 and I335 in the four systems. **(F)** The average distance between Y74 and I335 in the four systems; standard deviations are labeled in the histogram.

Since the aromatic nucleus of Y74 and methyl group of I335 can form one wall of the ammonia transfer channel in GPATase, the relative position variation analyses between two residues during aMD simulations were performed (Bera et al., 1999). The relative position variation analysis between I335 and Y74 were performed. The distances between Y74 and I335 of the four systems were distinctly different (Figures 11A–D). GPATase bonded to both cPRPP and DON had the shortest distance. I335 and Y74 formed one wall of the NH3 channel because the structure of the NH3 channel depends on hydrophobic amino acid side chains. Changes in the distance between Y74 and I335 during 400 ns aMD simulations are provided in Figure 11E, and the corresponding average distance and standard deviation are presented in Figure 11F. The distance of GPATase with DON and cPRPP remained stable at 6.2 Å, whereas that of GPATase with DON or cPRPP reached about 8 Å. The distance of the latter was not stable relative to that of the former. Therefore, DON and cPRPP played a role together to induce GPATase catalytic action.

The work of Kim *et al.* revealed that Y74 is an important residue for coupling between the Gln domain and PRTase domain by analysis of the x-ray structure model and the mutant enzyme (Kim et al., 1996). Subsequently, research from Bera et al. utilized fluorescence monitoring and determination of a K_d_ experiment to prove that Y74 and I335 were key residues for the interdomain signaling in GPATase (Bera et al., 1999). However, dynamic behavior changes of two residues induced by ligand binding remained unclear. Our dynamic analysis results demonstrated that the sole presence of DON or cPRPP in GPATase may disturb the interaction of these two key residues as the distance between them become larger, and only binding both DON and cPRPP maintains a close enough distance between them to have more interaction contacts. Thus it can be seen that DON and cPRPP functioned together for GPATase catalytic action.

The results of aMD simulations suggested that binding DON to GPATase affected the status and space of the PRTase flexible loop, and binding cPRPP had a great influence on the formation of a salt bridge in the glutamine loop of the enzyme. The salt bridge allowed contact between the glutamine domain and the PRTase domain and helped NH3 entry into the channel. These results were consistent with those of cMD simulations. The mechanism by which GPATase maintains stable states was clarified.

## Conclusion

GPATase catalyzed the synthesis of PRA from PRPP and glutamine at separate catalytic sites in different domains. In general, differences in conformations between active and inactive forms of GPATase occurred in flexible loops and extended to core domains. Moreover, binding PRPP to the PRTase domain depended on activating the reaction at the glutaminase domain, and the C-terminal reaction remained stable to keep the N-terminal in active state. Although binding cPRPP to the active site of PRTase could organize the flexible loop, only DON bonded to GPATase could retain the stable state of the loop and keep it closed. The glutamine domain and the PRTase domain approached each another when DON and cPRPP co-existed, a condition that is beneficial for enzymes to play catalytic roles. This study provides important dynamical evidence of conformational changes in DPEase for designing effective inhibitors that can target DPEase.

## Data Availability

The original contributions presented in the study are included in the article/Supplementary Material, further inquiries can be directed to the corresponding authors.
