# Supplementary material for: Conformational Changes of Glutamine 5′-Phosphoribosylpyrophosphate Amidotransferase for Two Substrates Analogue Binding: Insight from Conventional Molecular Dynamics and Accelerated Molecular Dynamics Simulations
Source: Front Chem. 2021 Feb 26;9:640994. doi: 10.3389/fchem.2021.640994 (PMC7953260; doi:10.3389/fchem.2021.640994)
Supplement: Supplementary file 1 [file DataSheet1.pdf]

Supplementary Material

Supplementary Figures and Tables

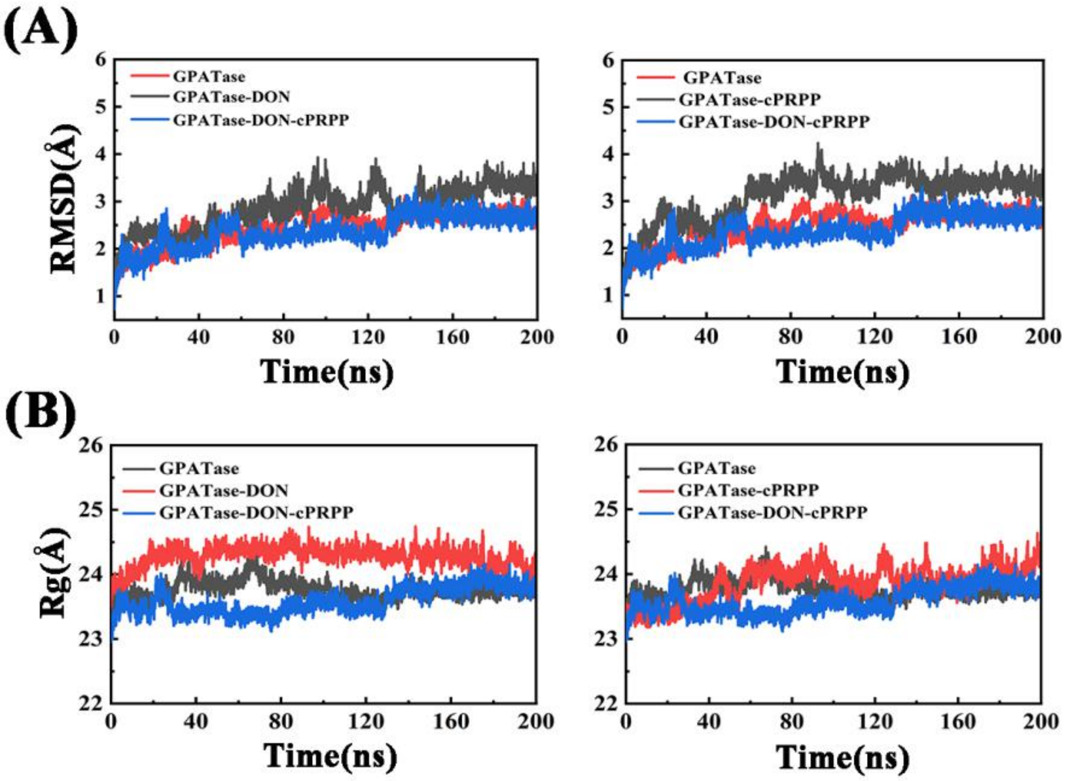

Figure S1. Stability analysis during 200ns cMD simulations for four systems. **(A)** The RMSD plot and **(B)** Radius of gyration(Rg) plot.

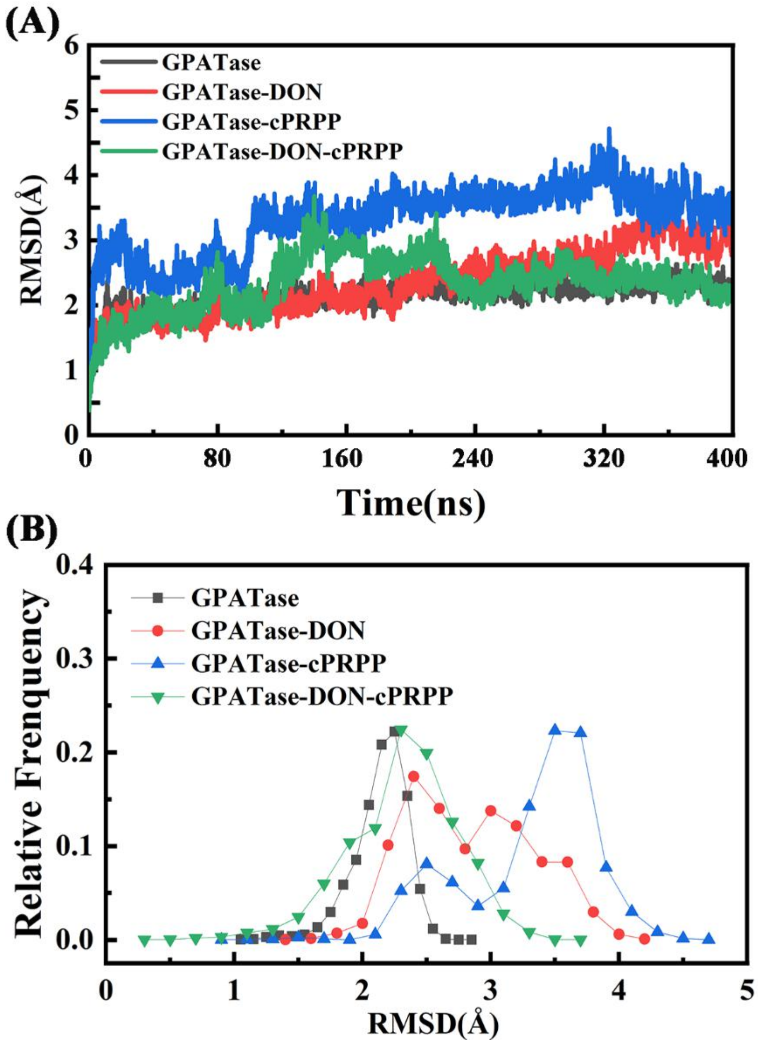

Figure S2. Stability analysis during 400ns aMD simulations for four systems. (A) The RMSD plot of Cα atoms. (B) The corresponding relative frequency RMSD values.

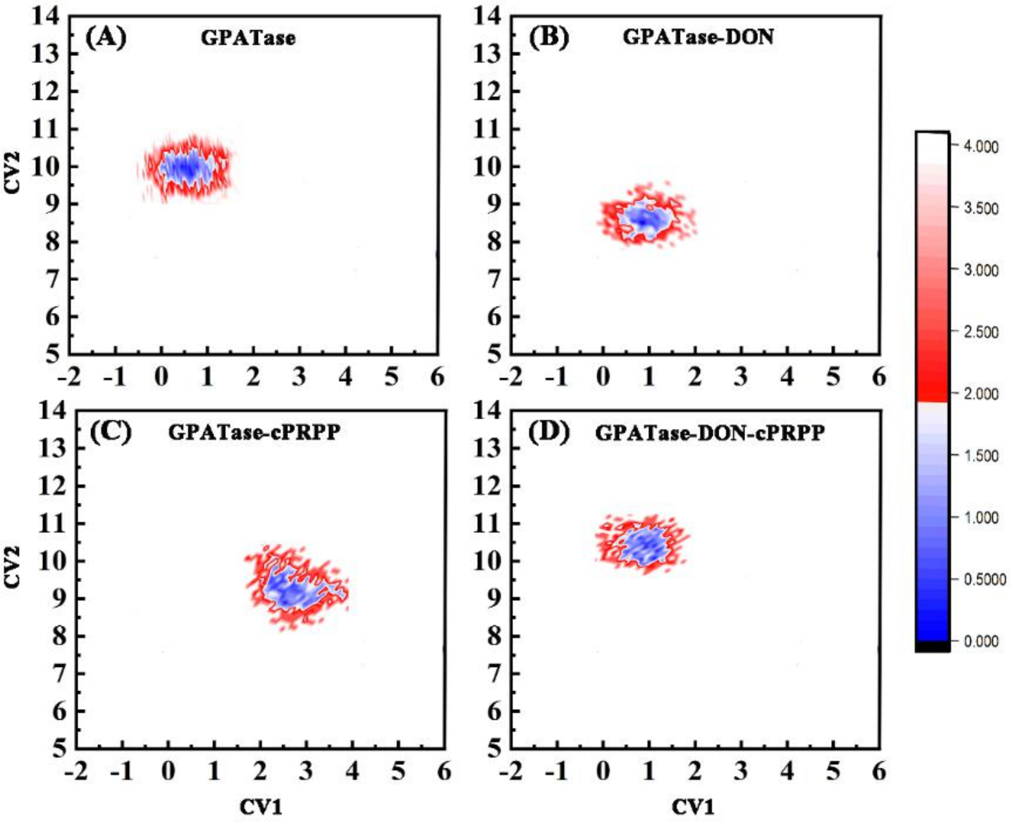

Figure S3. The free energy profile maps of flexible loops for **(A)** GPATase; **(B)** GPATase-DON; **(C)** GPATase-cPRPP; **(D)** GPATase-DON-cPRPP as a function of CV1 and CV2 in Å during cMD simulaitons.

Table S1. Clustering results for the top six clusters from GPATase model MD simulations trajectories with K-means clustering algorithm.

#Cluster	Frames	%	AvgDist[a]	Stdev[b]	Centroid[c]	AvgCDist[d]
**0**	6458	32.3	1.32	0.16	17321	2.12
**1**	3455	17.3	1.46	0.22	10827	1.95
**2**	2029	10.5	1.29	0.14	12286	1.95
**3**	1817	9.1	1.44	0.22	5837	1.69
**4**	1715	8.6	1.31	0.21	586	2.02
**5**	1277	6.4	1.30	0.20	2239	1.81

Table S2. Clustering results for the top six clusters from GPATase-DON model MD simulations trajectories with K-means clustering algorithm.

#Cluster	Frames	%	AvgDist[a]	Stdev[b]	Centroid[c]	AvgCDist[d]
**0**	3046	15.2	1.40	0.17	13644	1.32
**1**	2983	14.9	1.45	0.21	15864	1.41
**2**	2661	13.3	1.34	0.18	7767	1.53
**3**	2203	11.0	1.35	0.19	3133	1.78
**4**	2092	10.5	1.25	0.16	1500	2.14
**5**	1856	9.3	1.53	0.23	17478	1.58

Table S3. Clustering results for the top six clusters from GPATase-cPRPP model MD simulations trajectories with K-means clustering algorithm.

#Cluster	Frames	%	AvgDist[a]	Stdev[b]	Centroid[c]	AvgCDist[d]
**0**	4234	21.2	1.51	0.21	8412	2.06
**1**	3634	18.2	1.40	0.20	12680	2.00
**2**	2284	11.4	1.44	0.24	2355	2.16
**3**	2194	11.0	1.51	0.24	18376	2.19
**4**	1731	8.7	1.60	0.25	15455	1.79
**5**	1428	7.1	1.56	0.27	4250	1.99

Table S4. Clustering results for the top six clusters from GPATase-DON-cPRPP model MD simulations trajectories with K-means clustering algorithm.

#Cluster	Frames	%	AvgDist[a]	Stdev[b]	Centroid[c]	AvgCDist[d]
**0**	5109	25.5	1.36	0.19	11520	1.35
**1**	3248	16.2	1.49	0.23	7182	1.42
**2**	2854	14.3	1.32	0.16	17733	1.63
**3**	2579	12.9	1.37	0.20	14761	1.59
**4**	1721	8.6	1.40	0.20	16033	1.61
**5**	1372	6.9	1.35	0.23	1031	1.46

[a] AvgDist is the the average distance (Å) between all frames in the cluster

[b] Stdev is the standard deviation (Å) between all frames in the cluster.

[c] Centroid is the frame number of the lowest cumulative distance to any other frames.

[d] AvgCdist is the average distance (Å) of the cluster to any other cluster.
